# An Ounce of Prevention Is Still Worth a Pound of Cure, Especially in the Time of COVID-19

**DOI:** 10.5888/pcd18.200627

**Published:** 2021-01-07

**Authors:** Karen A. Hacker, Peter A. Briss

**Affiliations:** 1 National Center for Chronic Disease Prevention and Health Promotion, Centers for Disease Control and Prevention, Atlanta, Georgia

## The Chronic Disease Challenge Was Large and Is Now Larger

Before the coronavirus disease 2019 (COVID-19) pandemic, about 6 in 10 adults in the United States had a chronic condition; 90% of the nation’s health care expenditures were for people with chronic and mental health conditions, and chronic conditions accounted for 7 of the 10 leading causes of death in the United States (1–3).

The COVID-19 pandemic has only further emphasized the importance of chronic disease prevention and care — especially because many chronic conditions increase the severity of COVID-19 outcomes. For example, cancer, heart conditions, diabetes, and chronic kidney disease, in addition to being among the top 10 causes of death in the United States, are also established risk factors for severe illness from COVID-19 (4). A study from 12 states reported that 73% of people hospitalized for COVID-19 had at least one underlying chronic condition, and rates of hospitalization increased as the number of conditions increased (5). Additional chronic conditions or risk factors such as obesity and smoking also increase the severity of COVID-19 outcomes (4). COVID-19 may also contribute to social isolation and mental health challenges such as anxiety and depression (6). Finally, chronic diseases, risk factors for chronic disease, and COVID-19 all tend to disproportionately affect people of lower socioeconomic status and certain racial and ethnic minority populations. As a result, African American, Hispanic or Latino people, and American Indian and Alaska Native people are all at higher risk than non-Hispanic White people of getting sick, being hospitalized, or dying of COVID-19 (4).

Despite this relationship between chronic disease and COVID-19 and their related disparities, the pandemic has resulted in a decreased use of health services for emergencies and for ongoing preventive and routine health care. Although we cannot yet predict the effect of this trend on control of existing conditions, we do know that 4 in 10 Americans surveyed in June reported delaying or avoiding medical care during the COVID-19 pandemic and that delaying or avoiding care was even more common among people without insurance and among Black and Hispanic people (7). The extent to which control of chronic disease might mitigate a person’s COVID-19 risk is currently unknown, but we do know that appropriate management of chronic diseases such as hypertension, diabetes, and cancer saves lives. Needed care can be safely accessed, even during the pandemic.

Preventive care — screenings, vaccinations, mental health assessments, and oral and vision health — remain important even in the context of the pandemic. Vaccinations for preventing outbreaks of other infectious diseases (eg, measles, pertussis) are particularly important this year because childhood vaccination rates have decreased. Influenza vaccination is needed to reduce respiratory disease burden on an already taxed health care system. Finally, as they become available, vaccines against severe acute respiratory syndrome coronavirus 2 (SARS-CoV-2), the virus that causes COVID-19, will be a critical tool to help end the pandemic.

Throughout the COVID-19 epidemic, a great deal of anxiety has surfaced about safely accessing health care services. Health care providers have adopted a wide variety of strategies to mitigate risk. These strategies include measures to increase social distancing (eg, limits on waiting room capacity); mask wearing by all people in health care facilities; screening of patients for COVID-19 symptoms and, in some instances, for infection; and expanding the use of telemedicine. Patients now have a range of options for safely receiving care and should ask and be informed about these options when making appointments. Care that is needed acutely should not be delayed; vaccinations (for both children and adults) are essential services that should be given on time, and in-person nonurgent care (such as screenings) should be considered when risk of infection is low, based on local COVID-19 transmission rates, and when appropriate Centers for Disease Control and Prevention–recommended mitigation strategies are in place.

## Risk Factor Reduction Is Still Important, and More Can Be Done

Preventive care within the health care system is important, but risk reduction remains a mainstay for improving health and preventing disease. For example, tobacco use cessation is always important and perhaps even more so now given that tobacco product use is a risk factor for severe COVID-19 and that such use might plausibly increase risk of contracting COVID-19 (8). Resources for quitting can be accessed remotely and at no cost in many jurisdictions.

Regular physical activity can reduce risk of heart disease, type 2 diabetes, and some cancers. Particularly important at this time is that physical activity can also improve mood. People of all ages need to stay active to stay healthy. Although physical activities have sometimes been implicated in COVID-19 transmission — particularly when done in public, indoor, crowded, and poorly ventilated places (9,10) — numerous available strategies can make staying active safe (11).

Consuming healthy foods — including a variety of fruits and vegetables, whole grains, lean proteins, and low-fat dairy products — is another important component of health and wellness and can lower risk of obesity, type 2 diabetes, and heart disease.

Unfortunately, access to healthy foods has been a major challenge during the pandemic, particularly in low-income neighborhoods (12). Similarly, the availability of safe spaces for physical activity also varies by neighborhood. These conditions multiply the effect of other disparities and will require additional work to achieve short-term and long-term solutions.

## Relationships to the Social Determinants of Health

Issues such as limited economic opportunity, structural racism, and inadequate education have contributed to major health disparities in the United States, which the COVID-19 pandemic has only amplified (13). In turn, these issues drive current immediate challenges, including access to primary care, insurance coverage, paid sick leave, good housing conditions, and others.

These conditions, referred to as social determinants of health, exist where we are born, live, learn, work, and play and have a profound effect on health and our ability to narrow existing health disparities and those related to COVID-19. We will need to address these conditions to effectively provide opportunities to populations that have been historically disadvantaged. As we encourage people to reconnect to care and prevention, it will be imperative to address these social determinants of health. This work will require multiple partnerships within communities to reach out to people and rebuild trust in a safe and supportive health care system.

## What Additional Resources Do Health Care Systems and Communities Need?

As we seek progress against the pandemic, a backlog of critical chronic disease care will need to be addressed. This backlog could result from neglected emergent care, delayed preventive care and screening (eg, cancer screening), missed treatments (eg, cancer care, management of hypertension and diabetes), changes in health care access, and economic hardship. As just one example, it is estimated that COVID-19–related delayed screening and treatment of breast and colorectal cancers could result in almost 10,000 excess deaths in the United States (14).

The underutilization of these preventive services may have an even greater effect on people disadvantaged by preexisting disparities for whom issues, such as the effects of the pandemic on access to primary care practices and other health care settings and a lack of community resources, may be particularly acute. Telemedicine options may help streamline access to needed care, but these options depend on digital access, which is also likely to be most problematic for older people, low-income people, and people in rural areas.

To address these diverse challenges, providers and health systems need to ensure that patient communication is taking place to alert the population to the level of COVID-19 activity in the community, the risk and benefits of in-person visits, the available options for health care services (eg, telemedicine, in-person visits), and the current mitigation strategies for safe in-person visits. Providers and systems need to conduct outreach to populations with the greatest need for care and provide culturally and linguistically appropriate messaging that addresses historical mistrust and stigma.

## Conclusions

Chronic disease care has always been essential, and providing this care has become challenging during the COVID-19 pandemic, particularly as a result of delays in care and inadequate access. Now is the time to work together to improve awareness of the need for prevention and care and to encourage the safe utilization of needed care and the effective self-management of chronic diseases. Otherwise, we may find ourselves overwhelmed by poor health outcomes when the pandemic is over. This work will require innovative approaches from a health care system that must balance risks and benefits during the pandemic. Even more importantly, it will require a concerted effort to address the social determinants of health compounded by the COVID-19 pandemic that are driving today’s health inequities.
